# Priming of Attentional Selection in Macaque Visual Cortex: Feature-Based Facilitation and Location-Based Inhibition of Return

**DOI:** 10.1523/ENEURO.0466-19.2020

**Published:** 2020-04-07

**Authors:** Jacob A. Westerberg, Alexander Maier, Jeffrey D. Schall

**Affiliations:** Department of Psychology, Center for Integrative and Cognitive Neuroscience, Vanderbilt Vision Research Center, College of Arts and Sciences, Vanderbilt University, Nashville, Tennessee 37240

**Keywords:** attention, extrastriate cortex, pop-out, V4, vision

## Abstract

Visual search performance varies with stimulus and response history. Priming of pop-out refers to increased accuracy and reduced response time with repeated presentation of particular singleton and distractor features (e.g., a red target among green distractor stimuli), which are abruptly impaired when singleton and distractor features swap (e.g., green target among red distractors). Meanwhile, inhibition of return refers to the slowing of response time when target location repeats. Neurophysiological correlates of both these phenomena have been reported in the frontal eye field (FEF), an area in the frontal lobe contributing to attentional selection and eye movement planning. To understand the mechanistic origin of these adaptive behaviors, we investigated visual cortical area V4, an area providing input to and receiving feedback from FEF, during feature-based priming of pop-out and location-based inhibition of return. Performing a color pop-out task, monkeys exhibited pronounced priming of pop-out and inhibition of return. Neural spiking from V4 revealed earlier target selection associated with priming of pop-out and delayed selection associated with inhibition of return. These results demonstrate substantial involvement of extrastriate visual cortex in behavioral priming and inhibition of return.

## Significance Statement

Midlevel attention and visual processing is influenced by recent history of visual stimuli and gaze behavior. Using priming of pop-out visual search, we discovered that neural spiking in extrastriate visual area V4 shows speeded attentional selection when target and distractor features repeat and delayed selection when target location repeats. These neural processes paralleled but did not account for the magnitude of visual search performance changes with stimulus and response history. These new results improve our understanding of how recent experience influences attention and performance.

## Introduction

Repetitive performance of cognitive tasks often yields changing behavior. Priming is a salient example of this observation (Tulving and Schacter, 1990). Repeated performance of the same task can lead to behavioral improvements such as in pop-out visual search, where a target that differs in a single visual attribute has to be detected among an array of distractors. The priming of pop-out yields speeded response times and greater accuracy (Maljkovic and Nakayama, 1994). This effect has been replicated in monkeys and extended to show changes in neural processing (Bichot and Schall, 1999, 2002; Purcell et al., 2012; Westerberg et al., 2020). Electrophysiological concomitants of the priming of pop-out have since been shown in humans (Eimer et al., 2010).

The behavioral benefits at the heart of priming of pop-out are based on repeating the target feature (i.e., feature-based facilitation of return). However, not all forms of repetition yield behavioral improvements. Repetition of the target location yields slowed response times while maintaining a similar level of accuracy (i.e., inhibition of return; Klein, 2000; Taylor and Klein, 2000; Bichot and Schall, 2002; Fecteau and Munoz, 2005), albeit not in all instances (Maljkovic and Nakayama, 1996; Fecteau and Munoz, 2003). The neural mechanisms producing these behavioral changes is a matter of debate (Kristjánsson and Campana, 2010; Kristjánsson and Ásgeirsson, 2018). However, investigation into the neural sources of priming in visual search has provided crucial evidence.

The frontal eye field (FEF) and supplementary eye field (SEF) have been targets for neurophysiological investigations of priming in pop-out. While SEF neurons do not show modulation of activity with priming (Purcell et al., 2012; Westerberg et al., 2020), FEF neurons do (Bichot and Schall, 2002). Specifically, target selection (the time point where responses for the target stimulus diverge from that of distractors), measured in the activity of visuomovement neurons, is speeded with the priming of pop-out, while also being slowed following the repetition of target location. This result suggests that the priming of pop-out is based on the priming of attentional selection, which is also supported by studies in humans (Eimer et al., 2010; Brascamp et al., 2011).

FEF is not the only area implicated in priming of pop-out as lesions of V4 attenuate the behavioral improvements caused by priming (Walsh et al., 2000). FEF shares extensive connections with V4 (Schall et al., 1995; Stanton et al., 1995). V4 also shows robust modulation of activity with selective visual attention (Moran and Desimone, 1985; Reynolds and Heeger, 2009), and behavioral measures of selective attention are impaired following lesions of V4 (De Weerd et al., 1999, 2003). Furthermore, frontal feedback to V4 influences attentional modulation in V4 (Armstrong et al., 2006; Armstrong and Moore, 2007), although it is not entirely necessary (Gregoriou et al., 2014). However, the diversity of information being fed forward into V4 is arguably less than FEF. V4 has been shown previously to lag FEF in selection of the behaviorally relevant stimuli in search (Ogawa and Komatsu, 2006). This suggests that V4 is necessary for the manifestation of priming in FEF by supplying input of relevant information (e.g., features) but does not necessarily represent priming itself. Altogether, there exists anatomic and functional evidence supporting the hypothesis that attentional selection of V4 neurons modulates with the priming of pop-out; however, the alternative, that it does not, is also feasible.

Here we test the hypothesis that priming affects attentional selection in area V4. To do so, we recorded neural activity in V4 of two monkeys performing pop-out visual searches with embedded featural-priming and positional-priming sequences. To evaluate the similarities and differences between FEF and V4 during this task, we performed analyses identical to those of the prior study in FEF (Bichot and Schall, 2002). Specifically, we investigated (1) whether V4 neurons show target enhancement and distractor suppression with feature priming, (2) whether V4 target selection times are speeded with feature priming, and (3) whether V4 shows slowed selection times with repetition of target position.

## Materials and Methods

### Animal care and surgical procedures

**Figure 1.**
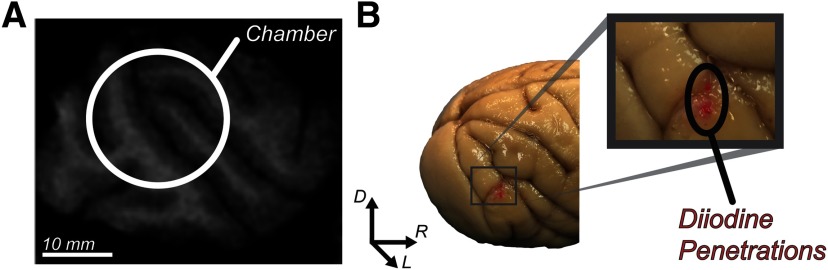
V4 localization. ***A***, 3 T structural MR scan of one monkey (right hemisphere, monkey *H*) with the position of the 19-mm-diameter recording chamber indicated by a white circle. The section is orthogonal to the 55° off-vertical axis of the chamber along which electrode penetrations were made. The plane of section is ∼23 mm from the midline. The lunate sulcus can be seen at the caudal edge of the chamber with the superior temporal sulcus running through the center. ***B***, Left, *Ex vivo* image of the posterior half of the brain of one monkey (monkey *H*). Right, Expanded view of a portion of V4 where two magenta dots can be seen. These dots indicate where the electrodes were placed (via dipping electrodes in diiodine) on the final two recordings in monkey H.

All procedures were performed in accordance with the National Institutes of Health Guidelines and the American Association for Laboratory Animal Care Guide for the Care and Use of Laboratory Animals, and was approved by the Vanderbilt Institutional Animal Care and Use Committee. Two male macaque monkeys (*Macaca radiata*; monkey C, 7.5 kg; monkey H, 7.3 kg) were implanted with a head post and recording chamber concurrent with a craniotomy positioned over area V4, which was located during a preoperative MR scan (Fig. 1*A*). Chamber placement was confirmed over V4 by a second, postoperative MR scan. Additionally, in one monkey, electrode penetrations were confirmed by staining two recording sites with diiodine before the animal was sacrificed (Fig. 1*B*). For chamber placement and craniotomies, monkeys were first tranquilized with ketamine (5–25 mg/kg) for intubation and catheterization before surgery. Surgeries were performed under aseptic conditions with the monkeys under and N_2_O/O_2,_ isoflurane (1–5%) anesthesia mixture. ECG, temperature, and respiration were monitored continuously throughout the procedure. Expired Pco_2_ was maintained at ∼4%. Postoperative antibiotics and analgesics were administered.

### Anatomical MRI

Monkeys were anesthetized using the procedure outlined under *Animal Care and Surgical Procedures*. Anesthetized animals were then placed inside an Intera Achieva 3 T MRI scanner (Koninklijke Philips) at the Vanderbilt University Institute of Imaging Science and remained anesthetized throughout the duration of the scan. Vital signs were monitored continuously. T_1_-weighted three-dimensional MPRAGE scans were acquired with a 32-channel head coil equipped for SENSE imaging. Images were acquired using a 0.5 mm isotropic voxel resolution with the following parameters: repetition time, 5 s; echo time, 2.5 ms; and flip angle, 7°.

### Experimental design and behavior

**Figure 2.**
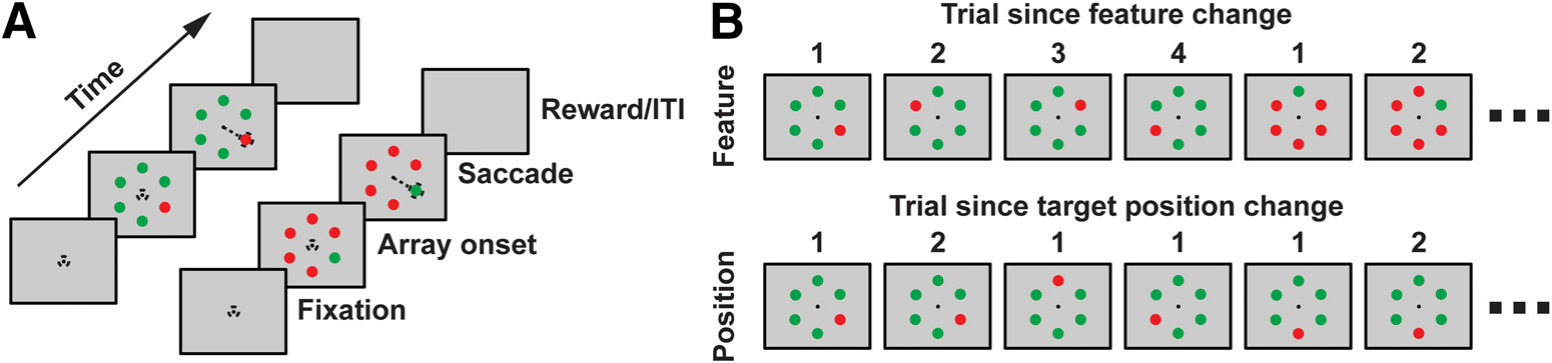
Behavioral task and trial sequences. ***A***, Order of events in a trial. Monkeys viewed a monitor where a fixation cross appeared at the center. After acquiring fixation and following a variable fixation period, a stimulus array appeared, consisting of a homogenous set of colored distractor disks and a single target of opponent color. Monkeys then were to saccade (denoted with a dashed line) to the target to receive a juice reward. Following reward, there was a short intertrial interval followed by the next trial. ***B***, Example trial sequences used for investigation of priming. Top, Feature priming, sequences of the same target feature (e.g., color, red vs. green) where “1” indicates the first trial where the target took on that feature. Numbers >1 indicate repeated trials of the same target feature. Bottom, Location repetition sequence where 1 indicates that the target appeared in a location different from that of the previous trial. Numbers >1 indicate a repeated target location.

Monkeys were trained to perform a pop-out visual search task presented on a CRT monitor at 60 Hz, 57 cm away, where the relevant feature was the color of a stimulus (Fig. 2*A*). We used the colors red and green for the target and distractor array items. These colors were rendered isoluminant and presented on a uniform gray background. Monkeys began a trial by fixating within 1° of visual angle (dva) around a central fixation cross. The amount of time between fixation acquisition and array onset was randomized and taken from a nonaging foreperiod function (Nickerson and Burnham, 1969; Naatanen, 1970, 1971), with times ranging from 750 to 1250 ms to reduce expectancy. Following the fixation foreperiod, a visual search array, consisting of six items was presented to the monkey. The size of the items in the array scaled with eccentricity at 0.3 dva per 1 dva eccentricity so that they were smaller than the estimated overall receptive field size (Freeman and Simoncelli, 2011). To determine the orientation and eccentricity of the array, each day online multiunit activity was measured during a receptive field mapping task (Cox et al., 2013, 2019; Dougherty et al., 2019; Westerberg et al., 2019), where the monkey fixated while a series of stimuli was presented across the visual field. The array was then oriented so that its eccentricity coincided with the location of the receptive field (3–10 dva eccentricity), and a single array item was placed at the center of the receptive field. Each trial, one randomized item in the array was of a different color than the others. To acquire juice, the monkey was tasked to saccade to that item within 1000 ms and to maintain fixation within a 2–5 dva space around the target for at least 500 ms. Eye movements were monitored continuously at 1 kHz using an infrared corneal reflection system (SR Research). If the monkey made the saccade to a distractor instead, the monkey did not receive a juice reward on that trial and experienced a short (1–5 s) time-out. Trials were presented in blocks of 5–50 trials with the vast majority of blocks being 5–15 trials in length (median length, 12 trials). The color of the target and distractors were held constant throughout each block (Fig. 2*B*, top). At the end of a block, the target color and distractor color were swapped. The target item had an equal probability of being located at any one of the six item locations. This led to a portion of sequential trials (∼16.67%) having the same target location. These sequences were used for investigation of inhibition of return (Fig. 2*B*, bottom).

### Neurophysiological procedure

**Figure 3.**
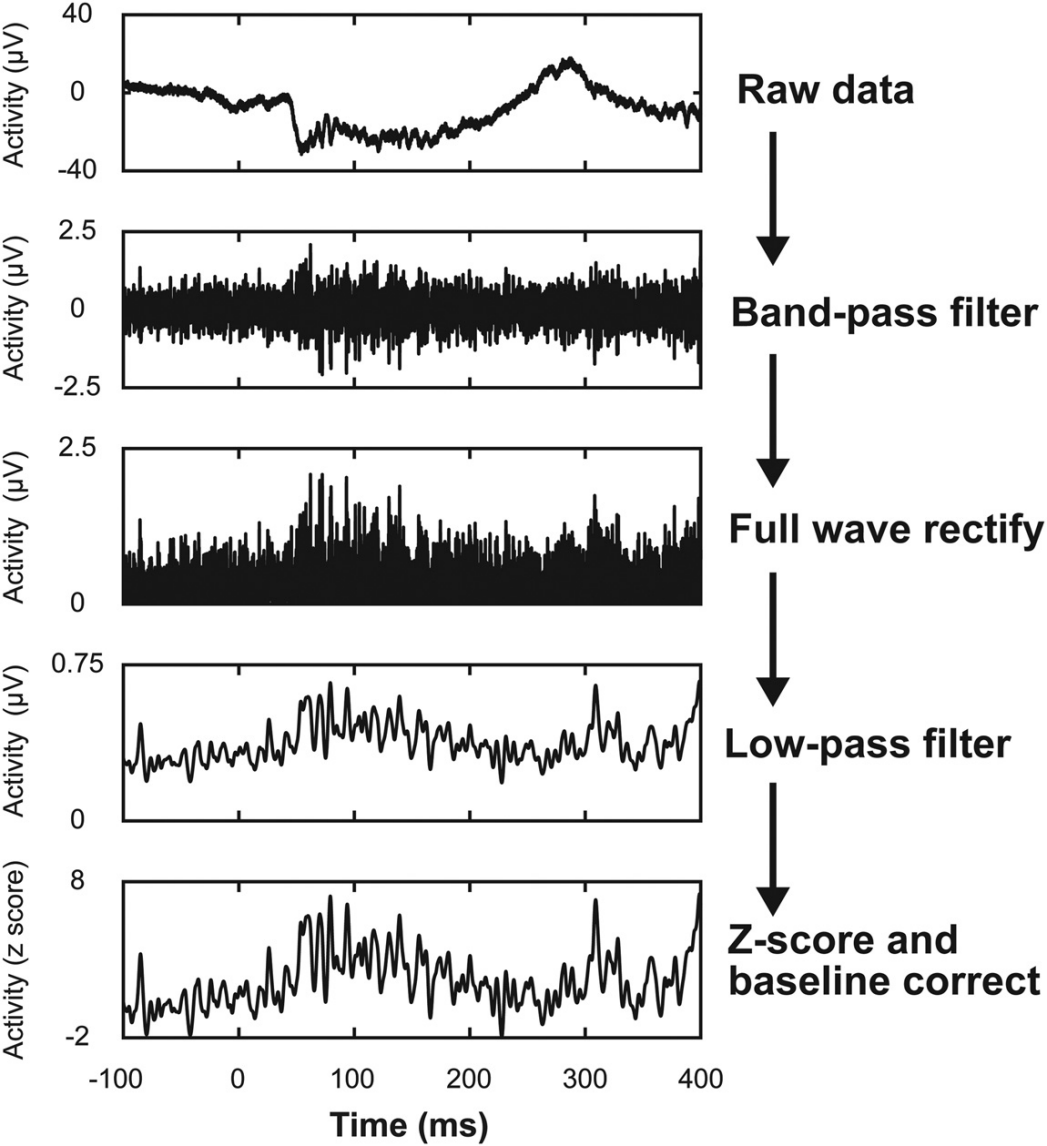
Derivation of spiking activity from raw data. Neural activity from each recording site was found by bandpass filtering the raw high-frequency (24,414 Hz) recording, then full wave rectifying, low-pass filtering, and finally converting to *z*-score and baseline correcting each trial. The results of each step in this procedure are depicted in descending order for a 500 ms window of time taken from a single trial from a session recorded in monkey C. Consequent analysis was then performed on the *z*-scored data.

Neural spiking data were acquired with 24 kHz resolution (Tucker-Davis Technologies) from V4 of both monkeys (left hemisphere, monkey C; right hemisphere, monkey H) using custom-variant 32-channel linear microelectrode arrays (S-Probe, Plexon) across 38 sessions (*n* = 31, monkey C; *n* = 7, monkey H). Spiking activity was derived from the multiunit activity measured at each electrode contact with a significant visual response to the array presentation (for further details, see Data analysis and statistics). This method for deriving population-spiking activity has been described previously (Legatt et al., 1980) and has been demonstrated to be effective across multiple brain areas (Logothetis et al., 2001; Roelfsema et al., 2004; Xing et al., 2009; Self et al., 2013; Shapcott et al., 2016; Tovar et al., 2019). Briefly, the broadband neural signal was low-pass filtered at 3 kHz, high-pass filtered at 300 Hz, full-wave rectified, and, last, low-pass filtered at 150 Hz (Fig. 3). This signal was used for the analysis of spiking activity throughout the study as multiunit activity has been shown to reliably reflect neural population dynamics (Trautmann et al., 2019) and disambiguation of visual and movement cell populations is unnecessary in V4, as was necessary in the previous study in FEF (Bruce and Goldberg, 1985; Bichot and Schall, 2002; Lowe and Schall, 2018). Additionally, multiunit activity in V4 has been shown to reliably reflect attentional modulation (Mehta et al., 2000; Nandy et al., 2017).

### Data analysis and statistics

Electrode recording sites in V4 were included for subsequent analysis if they had a significant response to the onset of the stimulus array and showed target selectivity. A significant visual response was defined as a change in the mean activity over the time between array onset and saccadic response that exceeded 2 SDs of the baseline activity (defined as the mean activity throughout 300 ms before array onset) for at least 50 ms of the array presentation epoch. Target selectivity was defined as a difference between a response to the target in the receptive field compared with the response to a distractor in the receptive field that exceeded 2 SDs between conditions for at least 25 consecutive milliseconds. A total of 469 visually responsive recording sites were identified, of which 201 (147, monkey C; 54, monkey H) showed target selectivity.

To compare the responses (e.g., response to target vs distractor, unprimed target to primed target), activity was normalized between the two compared conditions through a *z*-score conversion, as follows:

$$z_{t}=\frac{x_{t}-\text{mean}(x_{\text{baseline}\left(c1,c2\right)})}{s_{\left(\text{baseline}\left(c1,c2\right)\right)}}.$$

All trials were taken for the two conditions (c1 and c2) and transformed at each time point (t) into *z*-score ($z_{t}$) by subtracting the mean activity during the baseline across all trials encompassed by the two conditions ($x_{\text{baseline}\left(c1,c2\right)}$) being compared from the activity at any given time point ($x_{t}$), then dividing by the SD of the baseline epoch ($s_{\left(\text{baseline}\left(c1,c2\right)\right)}$). Because this normalization was condition dependent, the magnitudes of responses cannot be directly related across comparisons. For example, when comparing target and distractor responses for a given repetition (e.g., 1, 2, 3, . . . *n*), only trials of that repetition position were used in computing the normalized responses.

Target selection time was measured using previously reported methods (Bichot and Schall, 2002; but see Bradley et al., 1987; Britten et al., 1992; Westerberg et al., 2019), rooted in signal detection theory (Green and Swets, 1966; Macmillan and Creelman, 2009). We performed this analysis at the population level: using 1 ms increments, we compared the activity between the target and distractor conditions at each time point from 50 ms before array onset to 250 ms post-array onset by calculating receiver operating characteristic (ROC) curves. The area under the curve (AUC) quantifies the degree of separation between the conditions at each time point. An AUC of 0.5 indicates that the conditions are indistinguishable. As the AUC approaches 0.0 or 1.0, the conditions become more distinguishable. Therefore, plotting AUC as a function of time allows for evaluation of the discriminability of the target and distractor responses throughout the array presentation epoch. Finally, to derive a point in time where target selection can be compared across conditions, a cumulative Weibull function was fit to the AUC data across time, as follows:

$$P=\gamma -\left(\gamma -\delta \right)e^{-{\left(t/\alpha \right)}^{\beta }},$$

where P is the predicted AUC at time point t, γ, and δ are the maximum and minimum asymptotic thresholds, respectively, α is the time at which the distribution reaches 64% the maximum asymptotic threshold, and β is the slope. All parameters were free in the fits of all conditions. The exact time of target selection was taken as the point when the cumulative Weibull fit surpassed the midpoint between the maximum of the minimum asymptotic thresholds for the conditions being compared and the minimum of the maximum asymptotic thresholds. For example, in the comparison of target selection times for position in the feature priming sequence we found the minimum and maximum asymptotic values for all positions being compared. We then used the minimum of the maxima and maximum of the minima for those positions to determine the midpoint for computation of target selection time.

## Results

### Behavioral priming in pop-out visual search

Two monkeys were trained to perform a six-item pop-out visual search task. Sequences of trials in this task were organized in such a way that the target pop-out feature remained constant across blocks of trials. These blocks served to elicit feature-based priming of pop-out, as reported in other studies (Maljkovic and Nakayama, 1994; Bichot and Schall, 1999, 2002; Purcell et al., 2012; Westerberg et al., 2020). Briefly, monkeys fixated a central fixation cross for a variable amount of time before the appearance of the search array. The monkey then identified the color pop-out target (either a red target among green distractors or vice versa) by making a saccade to that item as quickly as possible. Successful responses were rewarded following a 500 ms target fixation period. Incorrect responses led to a short time-out and no reward.

**Figure 4.**
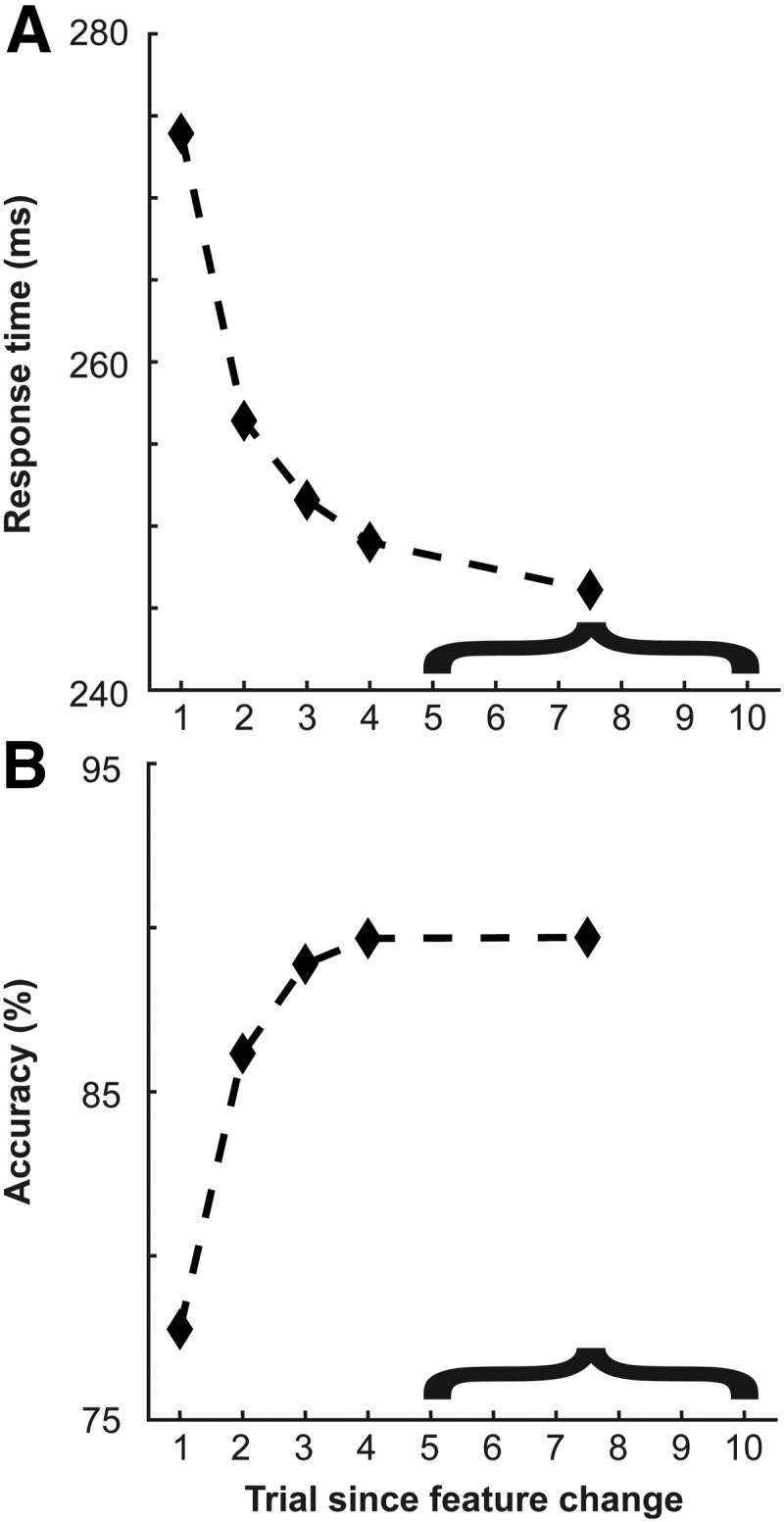
Priming of pop-out behavioral results. ***A***, Response time to target feature change across both monkeys (*n* = 2) and all sessions (*n* = 38). Trial 1 denotes when the target color changed from red to green or vice versa. Trials 5–10 were collapsed as the profile reached asymptote. ***B***, Accuracy as a function of trial since target feature change. Note that accuracy was always above chance (chance = 16.667%, 6 item array).

Both monkeys showed priming. Response times, measured as saccade latency, were consistently reduced through the priming sequences (Fig. 4*A*), reported here as trial from feature change (e.g., target changing from red to green or vice versa). Following a change in the target feature, performance degraded before improving again. Behavioral improvement seemed to show an asymptote around five trials following the feature change. For this reason, trials beyond this point were combined. The apparent change in response times was determined to be a statistically significant trend measured through Page’s *L* test (Page, 1963) on a session-by-session basis ($L_{38,5}=2033,p<<0.001$). This was also determined to be statistically significant in each monkey individually (monkey C: $L_{31,5}=1656,p<<0.001$; monkey H: $L_{7,5}=377,p<<0.001$). Accuracy improved through the priming sequences as well (Fig. 4*B*), as determined through Page’s *L* test on a session-by-session basis ($L_{38,5}=2006,p<<0.001$). This, too, was determined to be statistically significant in each monkey individually (monkey C: $L_{31,5}=1639,p<<0.001$; monkey H: $L_{7,5}=367,p<<0.001$). These results are consistent with previous reports of feature-based priming in humans (Maljkovic and Nakayama, 1994) and macaques (Bichot and Schall, 1999, 2002; Purcell et al., 2012; Westerberg et al., 2020).

### Speeded attentional selection in V4 with feature-based priming

**Figure 5.**
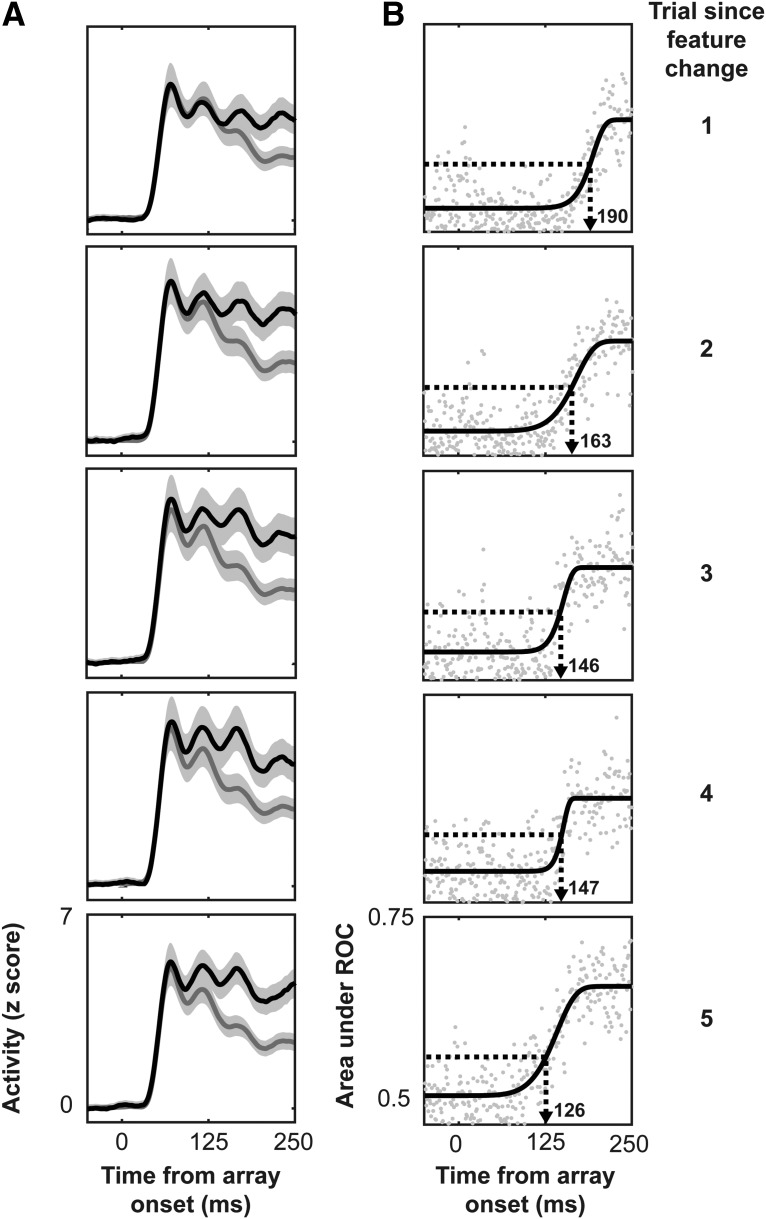
V4 target selection during priming of pop-out. ***A***, Spiking activity aligned on array presentation, averaged across all recording sites (*n* = 201) when the target was in the receptive field (black) versus when a distractor was in the receptive field (gray). Clouds represent 95% confidence intervals (CIs). Data are broken out by trial since feature change (right) from top to bottom. ***B***, AUC as a function of time from array onset where gray dots are the population average experimental data for each time point, and black curves are the cumulative Weibull fits. Computed target selection times (for details, see Materials and Methods) are indicated with an arrow pointing at the abscissa for each plot.

To determine whether V4 shows neural correlates of priming of pop-out, we measured target selection time across our neural population. Previous reports suggest speeded attentional selection may mediate behavioral priming in this task (Bichot and Schall, 2002; Eimer et al., 2010; Kristjánsson and Campana, 2010; Kristjánsson and Ásgeirsson, 2018) and V4 has been shown to be essential for attentional processing (for review, see Reynolds and Heeger, 2009). The neural measure of target selection time was adapted from methods of signal detection theory (Green and Swets, 1966). The details of how target selection times were computed can be found in the Materials and Methods. The threshold for target selection in ROC analysis was 0.5815. Spiking activity averaged across the population of target-selective V4 recording sites was divided up by (1) trial-since-feature-change and (2) whether the target or a distractor was present in the receptive field (Fig. 5*A*). We then computed the target selection time for the first five trials since feature change (Fig. 5*B*). We found that feature repetition decreased target selection time. Immediately following the switch of target feature, V4 neurons identified the target at 190 ms. This time decreased to 163 ms in the following trial before reaching the fastest selection time at 126 ms in the fifth trial following the feature change. To confirm that the change in target selection time was statistically significant, we performed a Page’s *L* test. To do so we subselected 80% of the recording sites and computed the target selection time, via the same ROC methods, 25 times. This method allows for the evaluation of the variability in target selection time across the population by repeatedly subsampling the data (Sato and Schall, 2003). We found a significant decrease in the target selection time of the whole population through these methods ($L_{25,5}=1363,p<<0.001$) as well as for the individual monkeys (monkey C: $L_{25,5}=1360,p<<0.001$; monkey H: $L_{25,5}=1172,p<0.05$). These results suggest that attentional selection of the target stimulus in V4 hastens with feature-based priming.

### Relationship between feature-based priming of behavior and selection in V4

**Figure 6.**
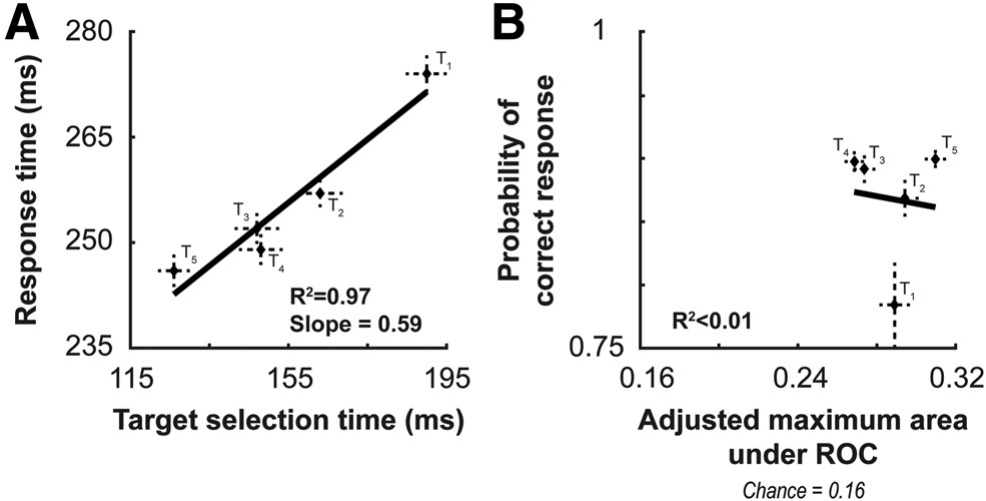
Effect of feature priming on the relationship between behavior and neural processing in V4. ***A***, Response time as a function of target selection time (see Materials and Methods). Dashed vertical and horizontal lines around each data point represent the 95% confidence intervals. The five points plot data from each of the five trials since feature change. Goodness-of-fit through adjusted *R*^2^ for the least-squares regression denoted in the bottom right. ***B***, Probability of a correct response as a function of adjusted maximum area under the ROC. Dashed vertical and horizontal lines around each data point represent the 95% confidence interval for probability of correct response and adjusted maximum area under the ROC, respectively. Goodness-of-fit through adjusted *R*^2^ for the least-squares regression is denoted in the bottom right.

We next sought to determine whether there was any correlative relationship between the changes observed in the behavior of the monkeys and the changes in target selection in V4, as previous work suggests that causal inactivation of V4 through lesions degrades the potency of priming effects (Walsh et al., 2000). We first plotted the relationship between changes in response time (averaged across sessions) against the population selection times (Fig. 6*A*). We performed a least-squares linear regression to obtain a fit and slope. We found a significant relationship, determined through a comparison against a constant model, between the behavioral and neural measures (*F*_1,4_ = 84.9; *p* = 0.0029). The slope of the fit, 0.59, was significantly different from 0 (*t*_3_ = 9.22, *p* = 0.0027). The slope measures the relationship between neural selection time and response time. If they changed perfectly proportionally (e.g., a 10 ms decrease in neural response time corresponded with a 10 ms decrease in response time), the slope would be 1. Slopes >1, as plotted, reveal a larger change in response time relative to the change in neural selection time (e.g., a 10 ms decrease in neural selection time corresponding to a 15 ms decrease in response time would yield a slope of 1.5). Slopes <1, but >0 reveal a smaller change in response time relative to the change in neural selection time (e.g., 10 ms decrease in neural selection corresponding to a 5 ms decrease in response time would yield a slope of 0.5). The slope measured here indicates that the variation in target selection time in V4 exceeds the concomitant change in response time.

While there appears to be a systematic relationship between the neural target selection time in V4 and behavioral response times, we did not find a relationship between the probability of a correct response and the magnitude of discriminability between target and distractor in V4 (Fig. 6*B*). The probability of correct responses was taken as the mean across sessions, and the magnitude of distinguishability was derived from the maximum AUC across time using the Weibull fit of the experimental data. This value was adjusted for the number of items in the array as ROC analysis is usually performed for two-alternative forced choice tasks while our arrays featured six possible choices. The conversion was done through a conversion table provided by Hacker and Ratcliff (1979; see also Smith, 1982; Macmillan and Creelman, 2009). The least-squares fit for the behavioral and neural measures of accuracy was not significant (*F*_1,4_ = 0.0097, *p* = 0.875), nor was the slope (*t*_3_ = −0.17, *p* = 0.874). In contrast to the change in performance as a function of position in priming sequence (Fig. 4), the discrimination of target and distractors in V4 was invariant across position in priming sequence. Hence, as observed in Figure 6*B*, the unprimed trial associated with the worst performance did not have the lowest adjusted area under the ROC.

**Figure 7.**
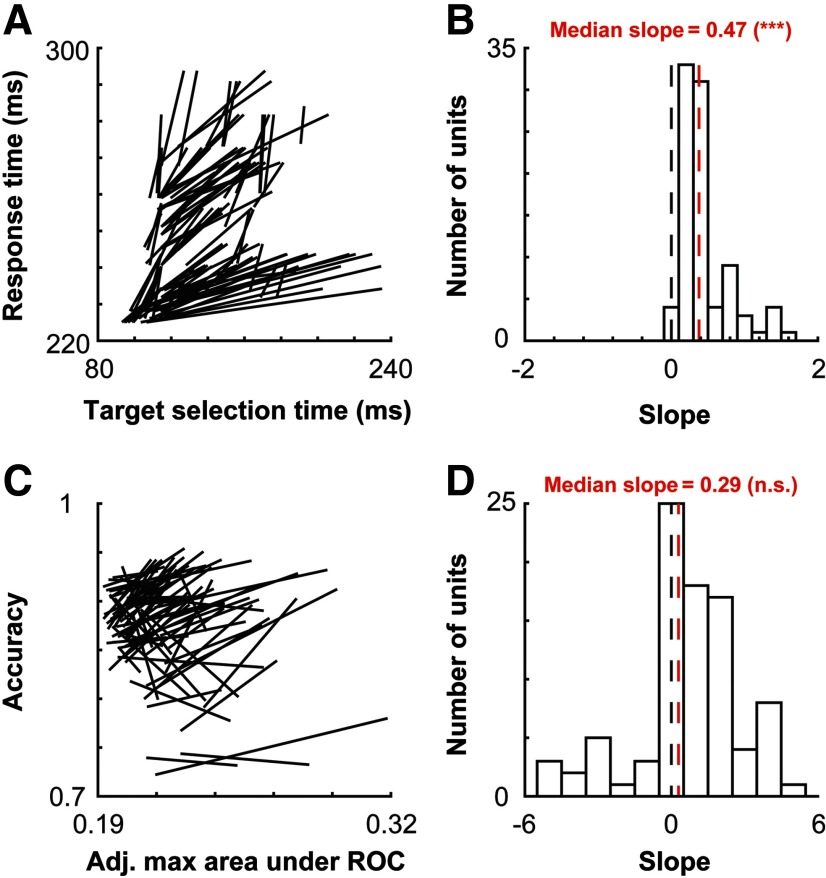
Relationship between neural and behavioral measures for individual units. ***A***, Linear regressions of neural target selection time and behavioral response time for each unit with a measurable change in target selection as a function of feature priming (*n* = 103). ***B***, Distribution of the slopes plotted in ***A***. The median slope of 0.47, which was significantly different from 0 (black dashed line, ***), is indicated by the red dashed line. ***C***, Linear regressions of adjusted maximum area under the ROC and behavioral accuracy for each of the units (*n* = 103). ***D***, Distribution of the slopes in ***C***. The median slope was 0.29, which was not significantly different from 0.

We then sought to determine whether these relationships between neural and behavioral measures could be found at the individual recording site level. To do so, we performed the Weibull fitting procedure (for details, see Materials and Methods) for each recording site as a function of trial since target position change. We then performed two linear regressions. One regression was performed between the measured target selection time of the individual recording site and the behavioral response time for the session (Fig. 7*A*,*B*). Another regression was performed on the measured adjusted maximum area under the ROC for the individual recording site and the behavioral accuracy for the session (Fig. 7*C*,*D*). Both regressions were performed as a function of the number of trials since the target feature change. Only units showing a significant difference in target selection time in the initial Weibull fit as a function of feature priming condition are shown in Figure 7. By bootstrapping the Weibull fits 25 times and performing an ANOVA (p<0.05) on the results for the target selection times as a function of trial since target feature change, of 201 units, 103 satisfied this criterion.

To quantify the relationship between the neural and behavioral measures, we measured the slopes for each of the regressions (Fig. 7). The median slope of the relationship between neural target selection time and response time was 0.47, which was significantly different from 0 (Wilcoxon signed-rank test: mean = 0.47; *Z* = 8.81; *p* = 1*e*^−18^). This number is <1 but >0, like the slope measured at the population level, indicating more variation over feature priming in target selection time than in response time. The median slope of the relationship between adjusted maximum area under the ROC and the behavioral accuracy was 0.29, which was not significantly different from 0 (mean = 0.29; *Z* = 0.38; *p* = 0.71). This parallels the finding at the population level, further indicating a dissociation between the discriminability by V4 units and behavioral accuracy.

### Target enhancement and distractor suppression during feature-based priming

**Figure 8.**
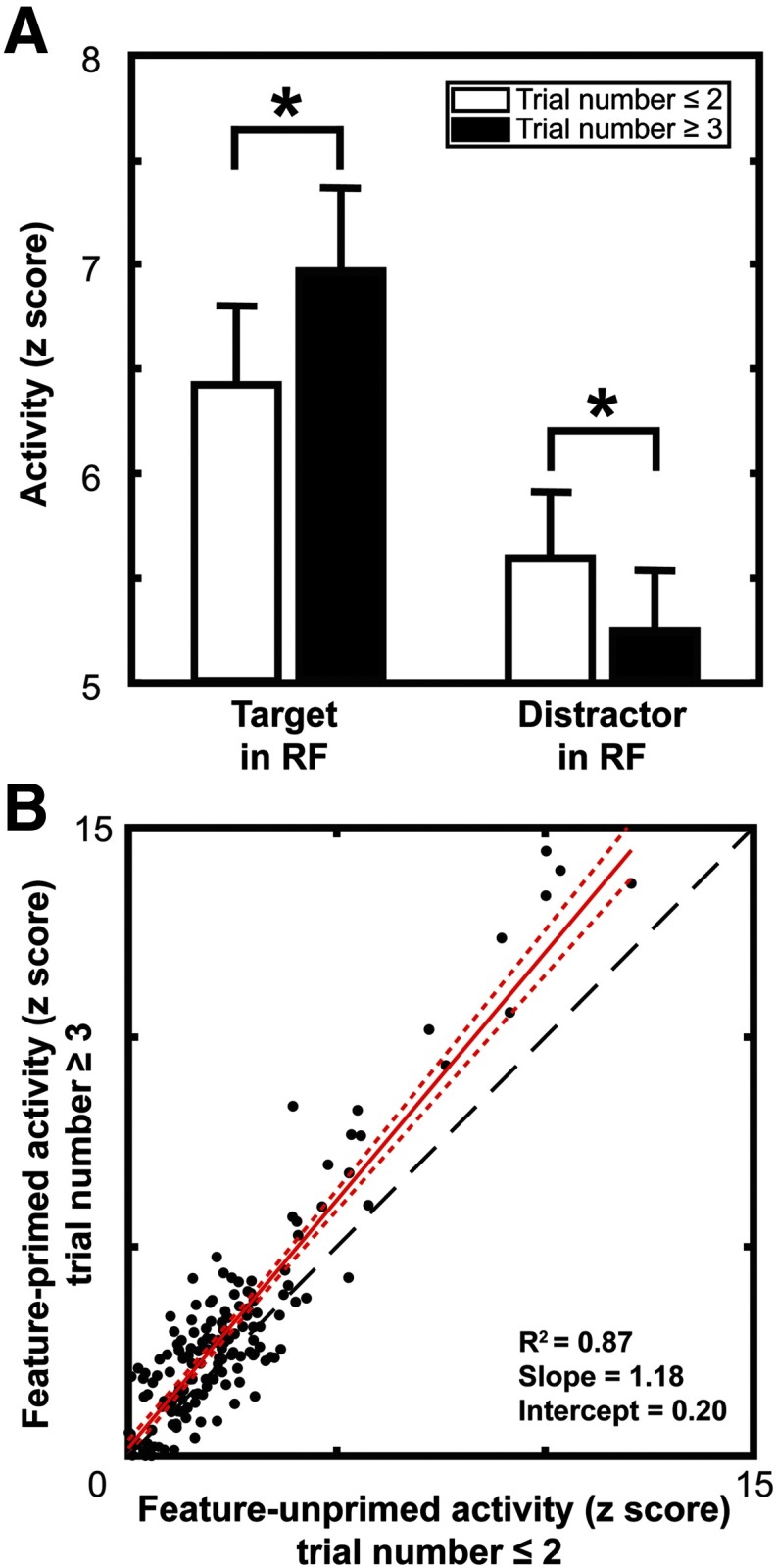
Target enhancement and distractor suppression during priming of pop-out. ***A***, Comparisons of target response magnitude (left) and distractor response magnitude (right) for trials early in the feature priming sequence (white bars) to trials later in the feature priming sequence (black bars). Error bars denote 2 SEMs. ***B***, Scatter plot showing the difference between target and distractor responses for each recording site. Unprimed activity on the abscissa and primed on the ordinate. Red solid line is the linear regression of the data with 95% CI represented by the red dotted line. Black dashed line indicates the one-to-one line. Values above this line indicate a greater difference between target and distractor responses in primed trials with points below the line indicating a larger magnitude difference in unprimed trials. The regression lies above that line, suggesting a greater target–distractor difference on primed trials.

Changes in attentional selection times could come about in a variety of different ways, such as enhancement of the target stimulus, suppression of distractors, or a combination of both. Previous work has shown that in FEF, both target enhancement and distractor suppression contribute to the effect (Bichot and Schall, 2002). We determined whether V4 shows target enhancement and distractor suppression with priming by comparing the mean spiking activity in the 50 ms preceding the average response time for early trials in the priming sequence to those later in the sequence (i.e., trials 1 and 2 since feature change and trial 3 or more, respectively). Results on the population level are shown in Figure 8*A*. We found a significant difference between the mean spiking activity in the unprimed target (mean = 5.91; SD = 5.58) and primed target (mean = 6.45; SD = 6.14) conditions ($t_{200}=-9.68,p=2e^{-18}$), which is in line with target enhancement during feature priming. Additionally, there was a significant difference between the mean activity in the unprimed distractor (mean = 5.09; SD = 4.70) and primed distractor (mean = 4.73; SD = 4.73) conditions ($t_{200}=7.15,p=2e^{-11}$), indicative of distractor suppression during feature priming. Both results were robust in each monkey (monkey C: target enhancement, $t_{146}=-10.85,p=2e^{-20}$; distractor suppression, $t_{146}=7.23,p=3e^{-11}$; monkey H: target enhancement, $t_{53}=-2.77,p=0.0077$; distractor suppression, $t_{53}=2.26,p=0.028$). Furthermore, we found no significant difference between the magnitudes of target enhancement (mean = 0.70; SD = 7.96) and distractor suppression (mean = 0.57; SD = 7.06) across the population ($t_{200}=1.41,p=0.16$).

We also performed a linear regression on the difference between target and distractor responses during the 50 ms preceding the average response time between the feature-primed and feature-unprimed trials (Fig. 8*B*). The regression significantly exceeds the unity line. This suggests a greater target–distractor response difference in the feature-primed trials. This is confirmed by the significant shift from zero of the intercept of the regression (intercept=0.20,p=0.033) and the slope being >1 ($\text{slope}=1.18,p=2e^{-91}$). These results parallel the findings in FEF and suggest that the changes in target selection times are mediated by a combination of changes in V4 processing of targets and distractors, rather than just one or the other.

### Baseline activity remains stable with feature-based priming

**Figure 9.**
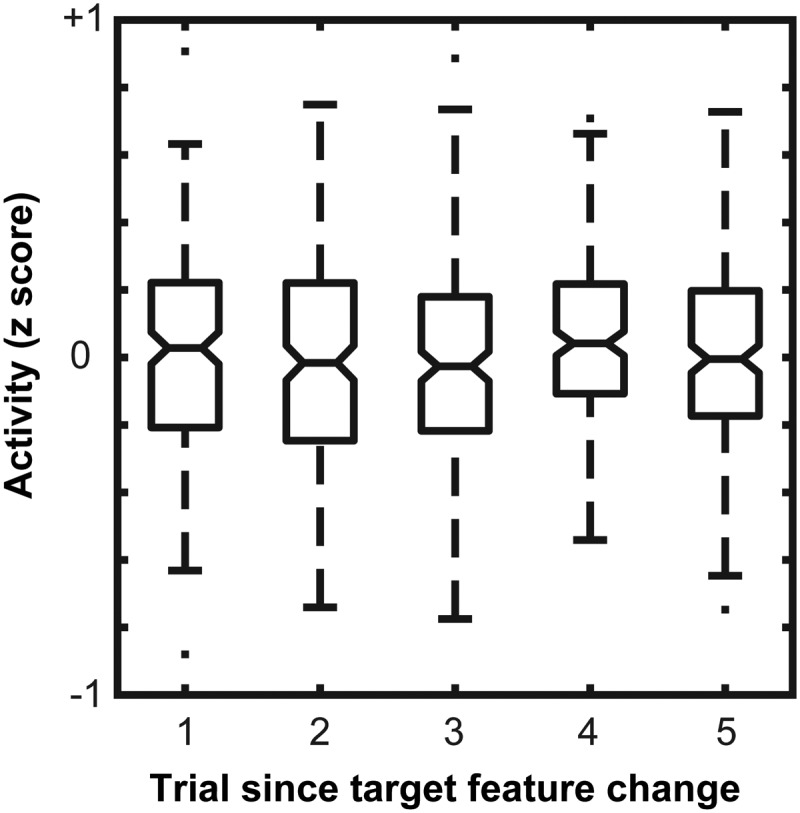
Baseline activity as a function of trial since target feature change. The center of each box is the average activity during the baseline for each condition. Horizontal line through box is the median. Upper and lower limits of the box represent the interquartile range. Whiskers represent the estimated minimum and maximum with dots outside those limits being potential outliers. Activity was measured as the average across all trials since feature change subtracted from each of the conditions. The baseline activity across trials since feature change remains consistent.

Spontaneous firing rates in area V4 have been shown previously to modulate with allocation of attention when locations are predictable (Luck et al., 1997). This sort of attentional modulation could account for the hastened target selection time observed in V4 with feature priming. By enhancing the spontaneous firing rate in V4 following a change in target feature, it is feasible that the selectivity for the feature is enhanced. While the monkeys are not cued to the location of the target stimulus before array onset in this task used in this study, we investigated whether baseline activity varied systematically following a change in target feature in the feature-based priming sequences. To do so, we measured the average neural activity in the 300 ms before presentation of the search array. Values were compared across the initial five trials in the priming sequence (Fig. 9). A one-way ANOVA revealed no significant difference (*F*_(4,1000)_ = 1.45, *p* = 0.21). Thus, the spontaneous firing rate in V4 during the baseline period does not change as a function of feature priming.

### Inhibition of return with target position repetition

The second form of repetition-induced behavioral modification that we were interested in is the changes associated with repetition of the target position in the search array. Previous work has shown two different behavioral outcomes under these circumstances. One is priming, where response times hasten when the target appears in the same location (Maljkovic and Nakayama, 1996). The other finding is inhibition of return, where repetition of target location leads to slowed behavior (Bichot and Schall, 2002; for review, see Klein, 2000). To determine the behavioral consequence of target position repetition in our task, we identified sequences where the target position changed and compared these with instances where target position was constant across subsequent trials. In our paradigm, the target had equal probability of appearing at any one of the six item locations that lead to repetition approximately one-sixth of the time, with sequences of three repetitions approximately one-thirty-sixth of the time.

**Figure 10.**
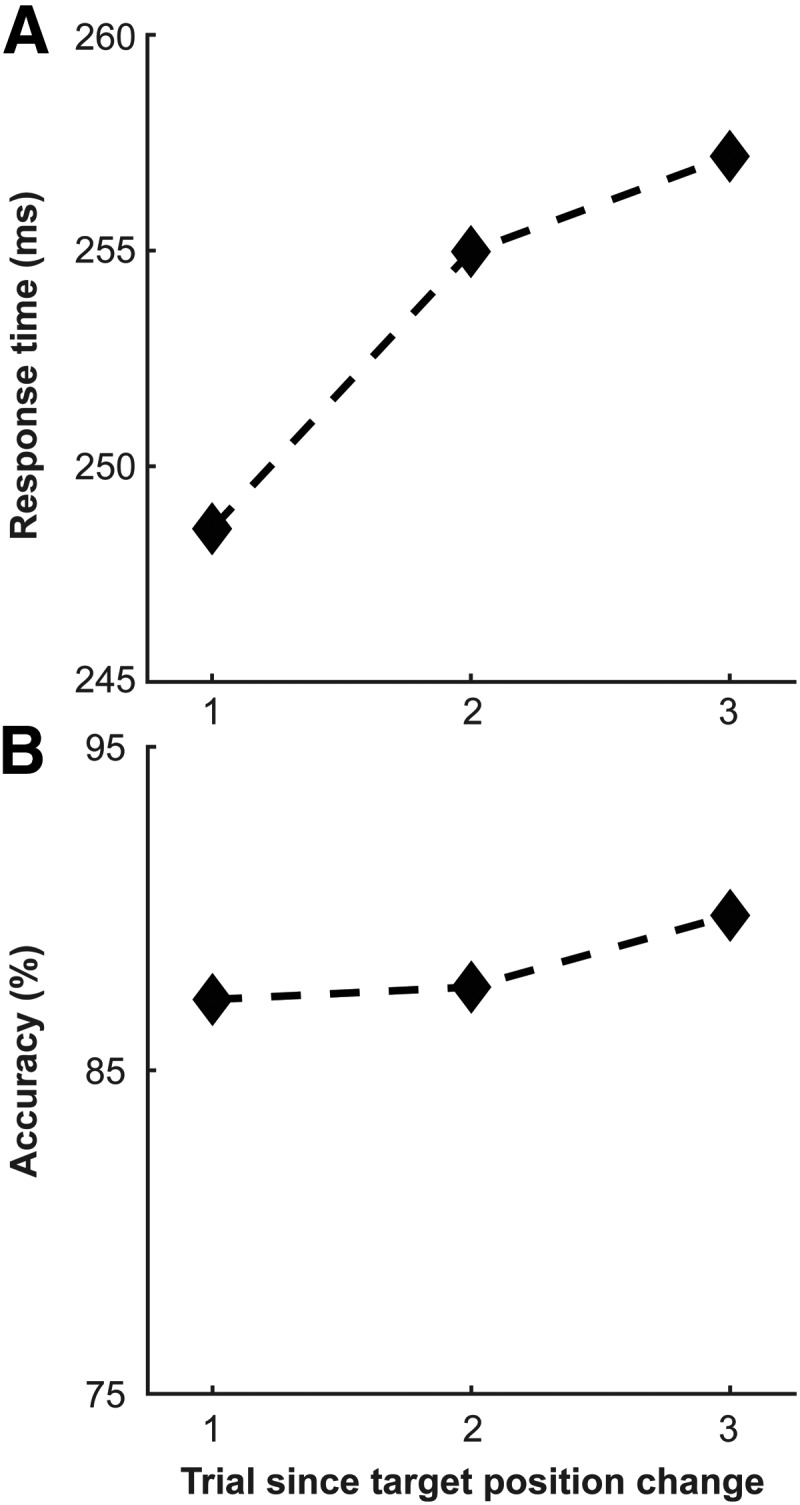
Behavioral results for target position repetition. ***A***, Response times as a function of trial since target position change across both monkeys (*n* = 2) and all sessions (*n* = 38). Trial 1 denotes when the target color changed from red to green or vice versa. Trials 5–10 were collapsed as the profile reached asymptote. ***B***, Accuracy as a function of trial since target position change. Note that accuracy was always above chance (chance = 16.667%, 6-item array).

Figure 10 summarizes the behavioral results of target position repetition. Qualitatively, it appears that response times increase with position repetition while accuracy remains largely unchanged. These results would replicate previous findings in monkeys performing this task (Bichot and Schall, 2002). Quantitative analysis tells a different story. Response time does significantly increase, as indicated by a Pages’ *L* test ($L_{38,3}=506,p<<0.001$), and this finding is robust for both monkeys individually (monkey C, $L_{31,3}=413,p<<0.001$; monkey H, $L_{7,3}=93,p=0.01$). However, while the increase in accuracy is small (0.43% from 1 to 2 and 1.53% from 2 to 3), it was a significant trend across both monkeys ($L_{38,3}=484,p=0.001$). However, when breaking out the data by monkey, it was only monkey C that showed this effect ($L_{31,3}=399,p<0.001$), while monkey H did not ($L_{7,3}=85,p>0.05$).

### Slowed attentional selection in V4 with target position repetition

**Figure 11.**
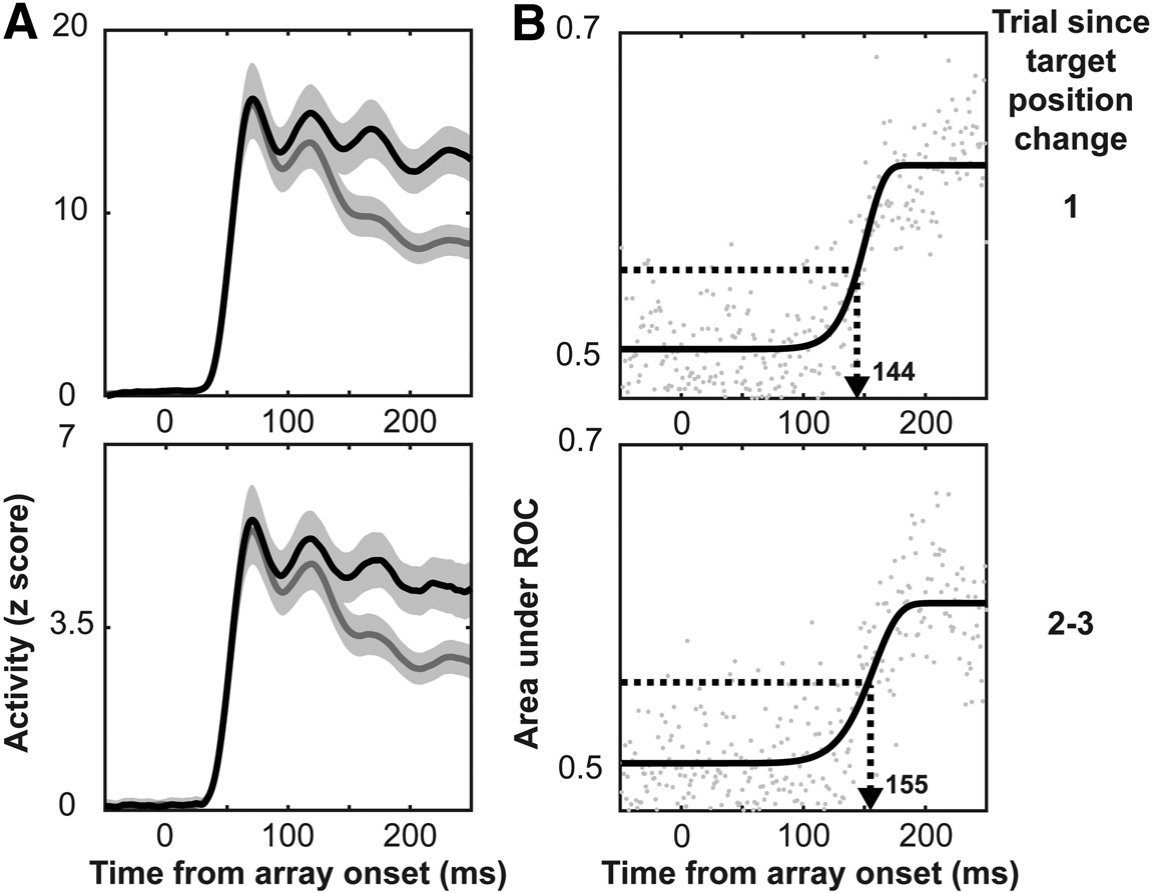
V4 target selection and location-based inhibition of return. ***A***, Spiking activity aligned on array presentation, averaged across all recording sites showing target selectivity (*n* = 201) when the target is in the receptive field (black) and when a distractor is in the receptive field (gray). Clouds represent 95% confidence intervals. Data are broken out by trial since target position change (right). ***B***, AUC as a function of time from array onset where gray dots are the experimental data and black curves are the cumulative Weibull fits. Computed target selection times (for details, see Materials and Methods) are indicated with an arrow. Data are broken out by trial since target position change (right) from top to bottom.

We next sought to determine whether neural correlates of inhibition of return were present in V4 as FEF has previously been shown to exhibit neural correlates of this behavioral phenomenon (Bichot and Schall, 2002). Evaluation of the neural correlates of inhibition of return in V4 was performed in a manner similar to that for feature-based priming. First, we plotted the spiking activity for each of the conditions comparing when the target was in the receptive field to when a distractor was present instead (Fig. 11*A*). We then evaluated target selection time as a function of trials since target position change using the same ROC methods. The threshold for target selection in the ROC analysis was 0.5703 for the analysis of target position repetition. We compared the trials where the previous trial had a different target location than the current trial (trial 1) to trials where the target had been in the same location for one to two previous trials (trials 2–3; Fig. 11*B*). We found that target selection times slowed by an average of 11 ms following target position repetition. This difference was found to be significant through a paired *t* test on the bootstrapped data where we subsampled 80% of the recording sites 25 times and computed the statistic ($t_{200}=-11.72,p=2e^{-11}$). This finding was confirmed at the individual monkey level (monkey C, $t_{146}=-16.83,p=8e^{-15}$; monkey H, $t_{53}=-6.29,p=2e^{-5}$).

### Relationship between location-based inhibition of return and selection in V4

**Figure 12.**
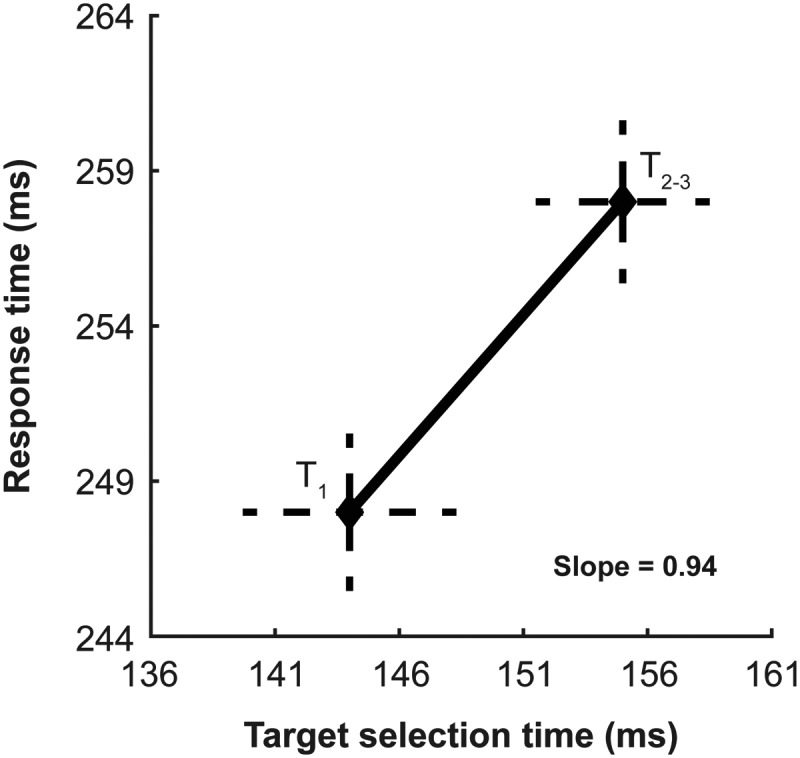
Effect of target position repetition on the relationship between neural responses in V4 and associated behavioral outcome. Response time as a function of target selection time in V4 measured through ROC (for details, see Materials and Methods) for trials where the target location was not a repetition and when it was. The two data points are the trial since target position change groupings from Figure 11. Dashed vertical and horizontal lines denote the 95% CIs around the means.

As we found a correlation between feature-based priming enhancements of behavior and target selection times in V4, we wanted to investigate whether a similar phenomenon could be observed with target position repetition. To do so, we plotted the mean response time as a function of target selection time in V4 (Fig. 12). As we had only two data points, we did not perform a least-squares fit. However, we nonetheless estimated the slope of the relationship by measuring the change in response times between the two points. We measured the slope of the line to determine the relationship between the change in selection time and response time. Again, a slope of 1 (a one-to-one relationship) would suggest that response time and selection time change at the same rate. Where the feature-based priming had a slope <1 between response time and target selection time, the inhibition of return condition did in fact have a slope near to 1. This suggests that as target selection times slow, behavioral response times increase at a similar rate.

### Target suppression with target position repetition

**Figure 13.**
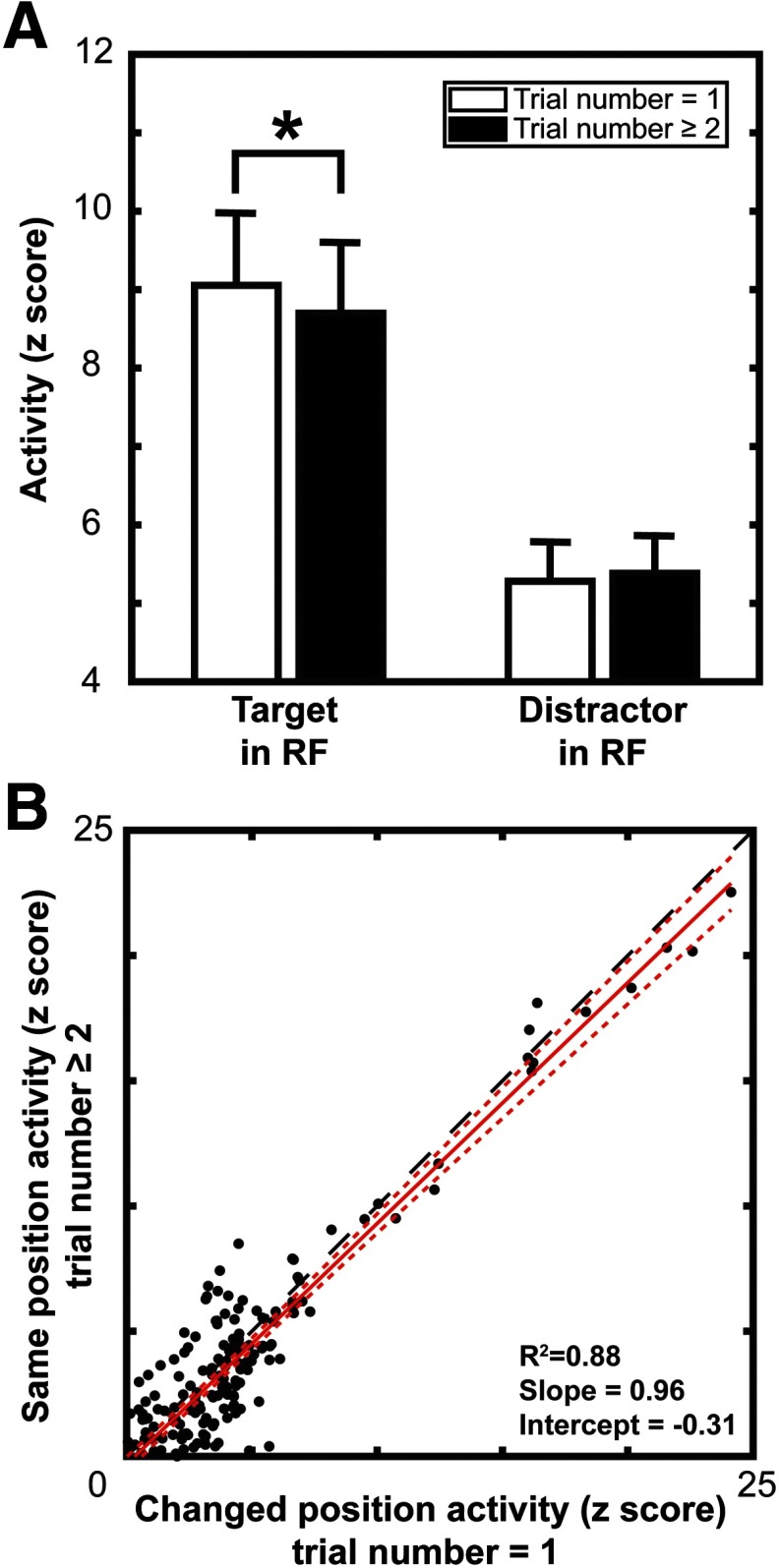
Target suppression with target position repetition. ***A***, Comparisons of target response magnitude (left) and distractor response magnitude (right) for trials where the prior target was in a different location (white bars) to trials where the target location repeats (black bars). Error bars denote 2 SEMs. ***B***, Scatter plot showing the difference between target and distractor responses for each recording site. Nonrepeat target location activity on the abscissa and repeated target location on the ordinate. Red solid line is the linear regression of the data with 95% CIs represented by the red dotted line. Black dashed line indicates the 1-to-1 line. Values above this line indicate a greater difference between target and distractor responses in target position repeat trials, with points below the line indicating a larger magnitude difference in target position change trials. The regression lies below that line, suggesting a greater target–distractor difference on target position change trials.

A slowing of attentional selection time can happen in several ways, such as suppression of the target, enhancement of distractors, or a combination of both. Hence, we determined whether V4 shows target suppression and distractor enhancement concomitant with inhibition of return. This was accomplished by comparing the mean spiking activity in the 50 ms preceding the average response time for trials in which the target position did repeat and when it did not repeat (i.e., trial 1 since feature position change and trial 2 or more, respectively). Results on the population level are shown in Figure 13*A*. Mean spiking activity was significantly different between the target position change (mean = 9.08; SD = 6.90) and the target position repeat (mean = 8.72; SD = 6.74) conditions for the target response ($t_{200}=4.60,p=7e^{-6}$), consistent with target suppression contributing to inhibition of return. Mean spiking activity was not significantly different between the mean activity in the target position change (mean = 5.30; SD = 3.81) and target position repeat (mean = 5.41; SD = 3.76) conditions for the distractor response ($t_{200}=-1.17,p=0.24$), indicating no contribution of distractor enhancement to inhibition of return. The target suppression result was robust in each monkey (monkey C, $t_{146}=5.04,p=1e^{-6}$; monkey H, $t_{53}=2.15,p=0.033$). Neither monkey showed significant distractor enhancement (monkey C, $t_{146}=-0.63,p=0.53$; monkey H, $t_{53}=-1.40,p=0.16$).

We also performed a linear regression on the difference between target and distractor responses during the 50 ms preceding the average response time between the target position change and target position repeat trials (Fig. 13*B*). Here we find that the regression line lies below the unity line. This suggests a greater target–distractor response difference in the target position change trials. This is confirmed by the significant shift from zero of the intercept of the regression (intercept=−0.31,p=0.037) and the slope measured to be below 1 ($\text{slope}=0.96,p=6e^{-90}$). These results suggest that the change in response time is mediated by a suppression of targets in the receptive field alone, rather than a combination of changes in target and distractor responses like that in the feature priming case.

### Baseline activity remains stable with target position repetition

While we did not observe a change in the spontaneous neural activity with feature-based priming, we sought to determine whether there was a change associated with target position repetition. To do so, we compared the baseline activity during the 300 ms leading up to array onset (while the monkey was fixating) of trials where the prior target position was outside the receptive field to trials where the target position repeated in the receptive field. Through a paired *t* test, we found no difference in the average activity between the target position change (mean, 0.0021; SD = 0.54) and target position repeat (mean = −0.058; SD = 0.62) conditions ($t_{200}=1.29,p=0.20$). This suggests that like feature-based facilitation, inhibition of return does not change the spontaneous firing rate in V4.

## Discussion

Behavioral priming can be elicited through feature-based facilitation of return in visual search under pop-out conditions. Previous work suggests that attentional priming mediates these changes (for review, see Kristjánsson and Campana, 2010; Kristjánsson and Ásgeirsson, 2018) as well as that frontal area FEF demonstrates neural correlates of these behavioral changes (Bichot and Schall, 2002). Noninvasive electrophysiological measures demonstrate associated changes in activity over posterior regions of the brain (Eimer et al., 2010), and a lesion study implicates visual cortex in priming (Walsh et al., 2000). To investigate whether visual cortex exhibits neural correlates of the behavioral changes observed in priming, we recorded population-spiking responses in area V4 of two macaque monkeys. We primarily sought to determine whether attentional selection in area V4 modulates with priming in feature-based priming of pop-out. We also wanted to determine whether neural correlates of location-based inhibition of return could be observed in V4 neurons. We found that attentional selection, measured as target selection time, modulated with both feature-based priming and location-based inhibition of return. These results suggest an explicit role for visual cortex in priming in pop-out and inhibition of return.

### Evidence for priming of pop-out as attentional priming

The mechanism underlying the behavioral improvements associated with priming of pop-out in visual search has been a matter of some debate in psychology (Kristjánsson and Campana, 2010; Kristjánsson and Ásgeirsson, 2018). While there is psychophysical evidence suggesting that priming of pop-out is mediated by memory mechanisms (Hillstrom, 2000; Huang et al., 2004; Huang and Pashler, 2005), electrophysiological evidence largely supports the idea that it is mediated through attentional priming (Bichot and Schall, 2002; Eimer et al., 2010). More recent psychophysical evidence also supports the attentional priming hypothesis (Brascamp et al., 2011). The findings of this study further support the idea that priming of pop-out is mediated through attentional priming mechanisms, as we found that the speed of attentional selection was speeded in V4 in this task. While it is possible that other cognitive phenomena, such as performance monitoring (Westerberg et al., 2020), may modulate as a function of priming in this task, they are likely secondary to the changes in attentional selection (Kristjánsson and Ásgeirsson, 2018).

### Putative circuitry for attentional priming

Few publications describe neural mechanisms of priming in pop-out visual search. FEF, a frontal area with known functional relevance to attention, eye movements, and vision shows speeded target selection during visual search (Bichot and Schall, 2002). SEF shows no changes during the period when attentional selection would occur (Purcell et al., 2012) and is therefore unlikely to contribute. Lesions of V4 and TEO suggest that those areas are necessary for priming effects (Walsh et al., 2000), and our results suggest that the speed of attentional selection is increased in V4 under these priming conditions. Together, these results suggest two plausible hypotheses regarding how attentional priming comes about in this task: (1) attentional selection is accomplished in visual cortex in pop-out visual search and repetition of target feature primes that selection, which is then inherited by FEF leading to shorter response times; and (2) attentional selection of the behaviorally relevant stimulus occurs in FEF, and the target selection signal is then fed back to visual cortex, leading to the observations in this study. To distinguish between these hypotheses, one could determine whether priming of pop-out and inhibition of return are evident in the feedforward input layers (granular layer 4) of V4 or only in the extragranular layers (layers 2/3 or 5/6). The vast majority of feedback to V4 from FEF occurs outside of the granular layers. Therefore, if the priming effects are first observed in the granular layers, it would suggest that these effects are generated in visual cortex. If the signals are exclusively observed in the extragranular layers, the history effects would either be from feedback to V4 or through processes occurring locally within V4. More definitively, one must record simultaneously from FEF and V4 and perform trial-by-trial analyses of target selection to determine the origins of the priming effect.

However, one hypothesis seems more likely given the quantitative results of these studies. In the study of FEF, there was a one-to-one relationship between the target selection time in that area and the response time across priming conditions. This indicates that as selection time decreased in FEF, the behavioral response time decreased equally as much. In V4, we observe a less than one-to-one relationship where target selection time decreased more strongly than response time. This relationship perhaps suggests that FEF receives crucial visual input from V4, but in resolving the behaviorally relevant stimulus, it does not perfectly integrate that information. This would mean that although V4 can more rapidly identify the target during priming, FEF acts as a bottleneck, in some manner. This combination leads to speeded responses, but not as speeded as would be predicted by the V4 activity. This hypothesis suggests that FEF target selection is necessary for generating the response but is not the sole origin of the behavioral effect.

The reason FEF does not speed target selection as rapidly as V4 may be because FEF receives input from many other visual cortical areas, which could add noise to the process (Schall et al., 1995; Stanton et al., 1995). The V4 lesion study discussed above perhaps provides some evidence for this as well (Walsh et al., 2000). Lesioning either V4 or TEO (both providing input to FEF) reduces priming while Lesioning either V4 or TEO (both providing input to FEF) reduces priming. Additionally, FEF shows a relationship between neural measures of discriminability and response accuracy, while V4 does not. This may suggest that V4 feeds FEF relatively invariant selection signals, whereas behavior more closely follows FEF neural measures of discriminability, which may be noisy, as previously suggested. It is important to remember that this notion is speculative, but it would suggest that attentional priming originates in sensory cortex before being inherited by the frontal area that then plans and initiates the response.

Further evidence for this hypothesis is available in comparing the measured target selection times in FEF previously and V4 now. Unfortunately, the FEF data are no longer readable, so this comparison is limited to published values. On the first trial in feature priming sequence, target selection time was earlier in V4 (190 ms; Fig. 5*B*) than in FEF (217 ms; Bichot and Schall, 2002). In subsequent trials, target selection times in V4 (trials 2–5: 163, 146, 147, and 126 ms; Fig. 4*B*) were somewhat later than those in FEF (trials 2–5: 131, 125, 117, and 120 ms; Bichot and Schall, 2002). These results seem reasonable in the context of the visual input filtering hypothesis for FEF target identification. We note that directly comparing the results of FEF to those of V4 may be unfair given that the data were obtained from different monkeys, which contributes unknown, subtle differences in behavior. Also, the summary times from Bichot and Schall (2002) were taken from a single representative unit rather than from the population. To draw more confident conclusions regarding how attentional priming comes about across cortical areas, simultaneous recordings or inactivation studies must occur.

The rationale behind this hypothesis lends itself to an interpretation of the inhibition of return result as well. While we found a less than one-to-one relationship with feature-based priming, we did find a one-to-one relationship between behavioral and neural processing for location-based inhibition of return. Whereas the priming of attentional selection may originate in visual cortex, this observation would suggest that V4 may inherit inhibition of return. Because of the task design (e.g., no predefined blocks of target position repetition, unlike the feature priming blocks), we are unable to determine whether this is a purely linear relationship as it may change further into the target position repetition sequence. Again, it is important to remember that these are hypotheses generated by the results of this study that must be tested more directly before a complete model of priming of attentional selection can be drawn.

### Comparative relevance

The finding of this study that priming of attentional selection is observed in V4, taken in consideration with the same observation in FEF (Bichot and Schall, 2002), may provide some insight into the nature of attentional selection signals observed through noninvasive measures in humans. More specifically, these findings may provide insight into the N2pc event-related potential (ERP) component, a measure of covert spatial attentional selection (Luck and Hillyard, 1994; Eimer, 1996; Woodman and Luck, 1999). The magnitude of the N2pc has been shown to be largest over occipital and parietal cortices (Luck and Hillyard, 1994), with evidence from a magnetoencephalography study showing potential sources of the N2pc in parietal and occipital cortex in humans (Hopf et al., 2000). Monkeys performing the same task show the same attentional selection signals in the ERP (Woodman et al., 2007) as well as similar, but earlier, concurrently recorded attentional selection signals in the spiking activity in FEF (Cohen et al., 2009; Heitz et al., 2010; Purcell et al., 2013). Together with the fact that FEF is extensively connected to V4 (Schall et al., 1995; Stanton et al., 1995), it is conceivable that FEF sends attentional selection signals to V4 where the N2pc is then generated. The priming results seem to corroborate this hypothesis. The N2pc in humans is speeded with feature-based priming in pop-out visual search (Eimer et al., 2010), as is attentional selection in FEF (Bichot and Schall, 2002). With the finding that FEF and V4 show speeded attentional selection, it is conceivable that V4 might be the generator of the N2pc that also shows speeded selection. Further work is needed to determine whether the biophysical properties of V4 could support the generation of the N2pc, however these functional correlates support the hypothesis. Further work is also necessary to determine whether FEF is necessary for the N2pc and whether it is necessary for speeding through priming or whether V4 alone is sufficient for this to occur.

## Synthesis

Reviewing Editor: Mark Laubach, American University Decisions are customarily a result of the Reviewing Editor and the peer reviewers coming together and discussing their recommendations until a consensus is reached. When revisions are invited, a fact-based synthesis statement explaining their decision and outlining what is needed to prepare a revision will be listed below. The following reviewer(s) agreed to reveal their identity: Behrad Noudoost. Note: If this manuscript was transferred from JNeurosci and a decision was made to accept the manuscript without peer review, a brief statement to this effect will instead be what is listed below.

One reviewer was from your submission to JN, and they would like you to address these points. The other reviewer and I agree that these issues should be addressed in a substantial manner in your revised manuscript.

1) I had suggested that the authors look into possible effects of priming on baseline activity in V4. Although the authors acknowledge that “The hypothesis that priming affects the baseline activity in V4 is interesting...” they respond that this is “...beyond the scope of this manuscript”. This is a simple analysis giving a simple answer to the question of whether or not priming affects V4 baseline responses. If the authors’ aim is to provide data on how priming affects V4 responses it seems to me that this analysis is not beyond the scope of this manuscript. After all, it is quite obvious that the authors fail to give a definite answer to what they initially claimed to be the aim of the study i.e. compare priming effects in FEF and V4 and find out where priming effects arise first. This is because of 1) the experimental approach (not using simultaneous recordings in the two areas in the same monkeys), 2) not being able to re-analyze the old FEF data because they are now unreadable (their response to the other reviewer) and 3) not being willing to analyze their laminar recording data to assess the origin of priming effects. Because of these, they have reduced the paper findings to a descriptive account of priming effects on V4 population activity. Adding a description of how baseline activity in V4 is affected by priming should thus be within the scope of this manuscript.

EDITOR’S COMMENT: This seems like a reasonable request. Please do address the issue of priming and baseline activity in V4 as suggested by the reviewer and include these results in the revised manuscript.

2) In my first review I had suggested that the authors provide some analysis on a unit by unit basis (namely multiunit by multiunit basis) rather than performing the ROC analysis on population average responses (their Figure 4). Apparently, the authors did not understand this point. They now provide a justification on why they used multiunit activity (which I agree is fine for the purposes of this paper) but not on why they did not perform the analysis for individual multiunits. To provide results similar to those of their older Bichot and Schall 2002 paper (Figures 2-5) it would be helpful to fit the data of each multiunit across trials with the Weibull distribution and show distribution of effects across all multiunits instead of bootstrapping the sample of multiunits to get estimates of population activity for the different trials in the priming sequence. At least for the feature priming analysis there should be enough trials for each multiunit to do that. This could help appreciate better the contribution of V4 neurons on feature priming effects and it would also make comparisons with the FEF easier.

EDITOR’S COMMENT: This seems like a reasonable request, especially in light of your earlier studies. Please fit the multiunits as described by the reviewer and include these results in the revised manuscript.

The other reviewer found some sections of the manuscript to be hard to read and suggested the following changes:

1) They need to make it clearer that the five points on figure 5 are 5 levels of “trial since feature change”. The sentence “Conditions match those of figure 4” is too vague.

EDITOR’S COMMENT: Please revise the text associated with Figure 5.

2) It is not a bad idea to bring figure 6 before figure 4. Figure 6 shows a classic phenomenon that V4 neurons differentiate target vs. distracters to begin with, and then in the same figure authors can build on that by emphasizing that the magnitude of target selection depends on priming and then in the next figure (current figure 4) show that indeed the timing of differentiation does also change.

EDITOR’S COMMENT: Please consider reordering the figures as suggested by the reviewer.

-3) Two suggestions to make the feature priming part of the paper less cryptic:

a) Add a sample neuron to the left side of current figure 4 with its raw firing rate. It is too much of a jump from task to a z-score version of the normalized firing rate. Walk the reader through a sample neuron as it is customary and then jump to a more sophisticated analysis.

EDITOR’S COMMENT: This suggestion and those below in the reviewer’s points 3b, 4, and 5 are all excellent suggestions that will improve the clarity of your results.

b) Add a scatter plot to figure 6. This is for your more expert audience always having a higher level of scrutiny. A scatter plot showing the z score difference between target and distracter in <=2 trials on x-axis and the same difference for >=3 on y-axis and will be a way more convincing way of data presentation for more critical minds.

4) For the location priming part: addition of a sample to figure 8, and clarification of what two data points on figure 9 indicate is also recommended.

5) What is clearly missing and maybe the only question I am adamant about it is the equivalent of figure 6 for the location priming dataset. Addition of a scatter plot similar to my suggestion for figure 6 is also recommended here.

6) I did not understand this sentence” “Where the feature-based priming showed a sub-1:1 relationship ...” Why not just calling a negative relationship. And BTW, I never understood why you report the slope in degrees in figures 5 and 9. It is a regression and you just report the regression values and their significance.

EDITOR’S COMMENT: Please provide comments on these textual issues and revise accordingly.
